# Examining Social Status Profiles with Gender, School Attended, SES, Academic Achievement and Wellbeing in Urban China

**DOI:** 10.1007/s10964-021-01454-8

**Published:** 2021-05-29

**Authors:** Wanying Zhou, Ros McLellan

**Affiliations:** Faculty of Education, University of Cambridge, Cambridge, UK

**Keywords:** Resource control theory, Social status, Latent profile analysis, Prosocial behavior, Adolescence, Wellbeing

## Abstract

Previous research has produced inconsistent findings about the relationships between aggressive and prosocial behavior with likeability and popularity. This study utilized latent profile analysis to identify naturally occurring social status profiles with these indicators and to explore their associations with gender, school attended, subjective social status, academic achievement, and wellbeing. The study recruited 818 (aged 12–15 years, 46% girls) Chinese adolescents and revealed four unique social status profiles: high aggressive-low likeability, low social status, average, and high prosocial-high social status groups. A bi-strategic profile did not emerge. The low social status and high aggressive groups exhibited the lowest academic achievement and wellbeing suggesting that more attention should be devoted to these students both in school and in future research.

## Introduction

Adolescents are most concerned about their peer social status during their crucial but sensitive middle school years where more complex social interactions occur in school but parental supervision is decreased (LaFontana and Cillessen, 2010). Some peer ecology and evolutionary-oriented researchers argue that prosocial and aggressive behaviors are two strategies which play similar functional roles in social dominance and the accumulation of social status, and that both can be exhibited by well-adjusted individuals (for a review see Pellegrini 2008). However, whether this so called bi-strategic group occurs naturally has not yet been established empirically, let alone whether such individuals are well-adjusted, as studies conducted in different countries have produced inconsistent findings (Berger et al., 2015; Hartl et al., 2020). Moreover, due to the heterogeneous nature of individual behavior and social status (von Eye and Bogat, 2006), variable-oriented studies have not come to a clear conclusion about the relationships between prosocial behavior, aggressive behavior, and social status (Lu et al., 2018a). With a cultural-contextual concern, this study was designed to confirm natural configurations of social status groups. In particular, it aimed to establish the existence of a bi-strategic group, and to explore status group associations with gender, school attended, subjective socioeconomic status, wellbeing, and academic outcomes, by deploying a person-centered analysis in a large sample of Chinese adolescents.

### Resource Control Theory in the Chinese Context

In Western literature, researchers generally concur that popular adolescents use both prosocial and aggressive behaviors to obtain and maintain their popularity (Cillessen, 2011); however, results in the Chinese context are less consistent. Some researchers have found that popular Chinese students exhibit similar patterns of aggressive and prosocial behaviors as their Western peers (Lu et al. 2018a, 2018b). However, others do not (Li et al., 2012; Owens et al., 2014). Thus, by shifting from a variable-oriented approach to a person-centered approach, the present research intends to grasp and interpret those associations from a different angle.

Resource Control Theory contends that prosocial behaviors and coercive behaviors are two evolutionary adaptations that are effective in securing and obtaining resources (Hawley, 2003). Considered as two functional strategies for social control, these two behaviors are believed to be socially adaptive and result in positive social outcomes (Hawley, 2014). Resource control theory classifies individuals into five groups: prosocial controllers, coercive controllers, bi-strategic controllers, non-controllers, and typical controllers, based on the distribution of self-reported behavioral strategies. In particular, the bi-strategic group has been found to score highly on prosocial and aggressive behaviors but enjoy a high social status in Western samples (Reijntjes et al., 2018).

A Chinese study, utilizing the same identification method as the studies cited above, testified to the existence of bi-strategic controllers in a non-Western sample but discovered that Chinese children in this group displayed poorer social functioning and lower peer status compared with their peers (Chen and Chang, 2012), a finding which stands in contrast to the assumptions of studies conducted in Western contexts. The categorization technique in the cited studies is based on a predetermined classification criterion of responses to locate participants into discrete groups. Although this method is person-oriented, it has been pointed out that it lacks the statistical ability to capture naturally existing profiles that don’t correspond to the predetermined statistical criteria (Berger et al., 2015; Hartl et al., 2020). Thus, the current study adopted latent profile analysis, which is a more advanced technique to avoid such methodological artefacts.

A small number of previous studies have used latent profile analysis to examine the natural occurrence of a distinct bi-strategic popular youth group; however, their outcomes are contradictory. A longitudinal study using aggressive and prosocial behavior, and popularity as social status indicators was able to identify this specific group in a Canadian student sample (Hartl et al.,^ 2020^). The bi-strategic popular group in this sample had the highest popularity, whereas the aggressive and prosocial popular groups had a similar level of popularity and the average group had the lowest popularity. Moreover, the bi-strategic group and the prosocial popular group had the highest peer acceptance and lowest loneliness. However, another cross-sectional study using aggressive and prosocial behavior, social status, and other socio-emotional indicators (e.g., perspective-taking, empathy) failed to confirm a bi-strategic group (Berger et al., 2015). In this study, a normative group, a high prosocial group, and a high aggressive group were detected; the aggressive group displayed the highest popularity, and the prosocial group showed the highest likeability. Researchers believe the reason for the contradiction was that the latter study featured a broader array of indicators, which in turn exposed different profiles (Hartl et al., 2020). For this reason, the current study narrowed the range of indicators focusing on resource control elements, which only identify prosocial behavior, aggressive behavior, likeability, and popularity, to test if these two sets of behaviors could co-exist in a social status profile.

Beyond different indicator choices, cultural difference could be another factor that impacted the aforementioned inconsistent findings since the functional meanings and outcomes of different behavioral patterns are profoundly impacted by cultural context (Chen and French, 2008). Practices of aggression and conflict are unwelcome, and the virtues of tenderness and kindness are more widely accepted in a collectivist environment (Cowell et al., 2017). Furthermore, in Chinese schools, moral education is emphasized to teach students to maintain harmony in classes and to be altruistic (Chen et al., 2000). Such culturally endorsed values signify that Chinese-identified profiles and the outcomes of profiles might differ from those in Western domains. Accordingly, the current study aimed to ascertain whether the bi-strategic group naturally exists in a collectivist cultural context and, if so, whether the outcomes are adaptive as proposed in resource control theory.

### The Indicators of Social Status Profiles

In previous decades, two indicators of social status, namely social preference (likeability) and perceived popularity (popularity), were traditionally used to distinguish two types of social status in peer relationships (Cillessen, 2011). However, researchers use different terms (e.g., sociometric popularity, perceived popularity, popular, or social status) interchangeably, meaning that studies on this topic are hard to clarify and compare (Cillessen and Rose, 2005). To ensure greater clarity, this study applied likeability to reflect peer acceptance and social preference, while popularity was adopted to describe youth who are visible, prestigious, and dominant among their peer groups. Furthermore, social status is used in this paper as an umbrella term to encompass both types of indicators (likeability and popularity).

The relationship between aggressive behavior and social status is currently unclear. On the one hand, aggressive behaviors are historically seen as maladaptive (Findley and Ojanen, 2013), which leads to young people who demonstrate such behaviors having low likeability. On the other hand, aggressive behaviors make certain young people more visible and more potent in their classes (Cillessen and Mayeux, 2004). Given likeability and popularity are two aspects of social status, the relations between aggression and social status as a whole are hard to reconcile. This complicated relationship necessitated the separation of social status into two dimensions (likeability and popularity) in this study. Aggression comprises indirect aggression and direct aggression (physical and verbal aggression). Indirect aggression also refers to relational aggression which involves social or peer relation harm. Physical aggression describes actual physical harm, and verbal aggression refers to verbal confrontations or making fun of others (Archer, 2004). Moreover, aggressive behavior has been discovered to be negatively associated with academic achievement and wellbeing (Rodkin et al., 2000). As the two types of aggression display significant gender differences (Forbes et al., 2009), the current study used both physical and relational aggression as profile indicators.

Empirical studies involving both Chinese and Western students tend to reach the same conclusion that prosocial behaviors are positively associated with both likeability and popularity. Prosocial behaviors which are defined as voluntary and beneficial actions to others are universally welcomed in school settings. In contrast to aggressive students, students who engage in kind and assisting conduct are more likely to have better wellbeing and learning outcomes (Caprara et al., 2000). Additionally, cooperation behaviors are much more frequently seen in China than in the US as Chinese peer relationships entail undertaking a large number of collaborative activities in the pursuit of group coherence (Chen and French, 2008). Thus, the current study included two forms of prosocial behavior, cooperation and helping behavior, as profile indicators. Taken together, the following six indicators were used in the identification of profiles: help, cooperation, relational aggression, physical aggression, likeability, and popularity.

### Self-Perceived Socioeconomic Status, School Type, and Gender

Several studies have shown that a lack of socioeconomic resources predicts a lower level of social competence and emotional wellbeing, and a high level of social and behavioral difficulties in schools (Bierman et al., 2008). As the perception of socioeconomic status is more important than reality in predicting quality of life (Netuveli and Bartley, 2012), Subjective Social Status (SSS), which captures a person’s sense of their place within a hierarchy (Adler and Stewart, 2007), was used in the current study to examine whether students’ perception of their socioeconomic status predicts their profile memberships. To date, very few studies have looked at the impact of school type (public vs private) in China. In general, students who attend fee-paying private schools have higher socioeconomic backgrounds than students in public schools (Shi et al., 2017). Private schools usually have smaller class sizes and better teaching resources and facilities which may create a different class culture including peer relationships compared to public schools (Wen et al., 2008). Thus, the current study also examined school type on the membership of social status profiles. Regarding gender, both variable-centered studies and person-centered studies concur that boys are more likely to behave aggressively than girls (Salmivalli and Kaukiainen, 2004). Findings from two latent profile analysis studies are consistent; boys are more likely to be in the aggressive popular group and girls are more likely to be in the prosocial popular group (Berger et al., 2015; Hartl et al., 2020). Thus, the current study also tested the extent to which gender is associated with the membership of social status profiles.

### Academic Achievement and Wellbeing

A Chinese study has found academic achievement was positively associated with both likeability and popularity (Niu et al., 2016), while Western studies have claimed no such relationship (Meijs et al., 2010). The inconsistent results may be explained by the norms of academic achievement in different countries (Niu et al., 2016); academic high achievers in Western culture may be treated as “nerds” or “outliers” in their classes (Li, 2007), whereas given that there is a strong academic-centered orientation in Chinese schools (Hau and Ho, 2010), this type of student is normally favored by their peers in the Chinese context. Furthermore, students in China with better scores are more likely to be assigned leadership positions and be expected to offer academic support to classmates (Li et al., 2012). One empirical study found the level of prosocial behavior is the prime predictor of academic achievement five years later, even when controlling for primary academic grades (Caprara et al., 2000). The current study thus examined the relationship between membership of social status profiles and academic achievement.

To better facilitate different social status groups, it is crucial to gauge the impact of different social status groups’ memberships on the wellbeing of students. Neuroscientists have found that a top-down neural control directly linked a prosocial decision to an increase in happiness (Park et al. 2017). Moreover, social science researchers have noted that fostering social relations might be another explanation for why engaging in prosocial behaviors can produce wellbeing (Diener and Seligman, 2002), since strong social relationships are vital to maintain wellbeing. Little is known about the relationship between social status and wellbeing. There has only been one correlational study that has directly investigated this and it found that the relationship between social status and social contentment is non-linear (Ferguson and Ryan, 2019). A previous study utilizing clustering analysis found prosocial groups displayed the lowest level of anger and disruptiveness (Hartl et al., 2020), but no prior studies have looked at each group’s wellbeing or academic achievement. Thus, this study assesses academic achievement and psychological wellbeing and investigates their relationships with social status profile membership.

## Current Study

Building on previous research, the present study shifted from examining relationships between behavior and social status through a variable-centered approach to understanding how these intercorrelated variables are displayed in different people. The first goal of the current investigation was to identify social status profiles among Chinese adolescents based on the narratives of resource control theory (prosocial behaviors, aggressive behaviors, popularity, and likeability); and specifically, to investigate whether a bi-strategic group exists. Based on the literature and taking cultural aspects into consideration, a high prosocial social status group with high prosocial behavior and high social status, and an average group with some social status were expected. However, the bi-strategic group was not expected to appear in Chinese adolescents. Last, it was hypothesized that there would be an aggressive group exhibiting low likeability, but their popularity level could not be predicted. Further, relatively little attention has been given to the prognostic factors and the implications of being in the different social status profiles. Thus, a unique feature of the current study was to examine how these different profiles relate to gender, school attended, self-perceived SES, academic achievement, and wellbeing in a Chinese middle school context. It was anticipated boys would be more likely to fall in the aggressive popular group and that students who have high subjective social status would have a higher chance of being in the prosocial social status group. Moreover, it was anticipated that students from the prosocial group would have higher levels of academic achievement and wellbeing, but, in contrast, the aggressive group was expected to have a low level of academic performance and wellbeing.

## Method

### Participants

Participants comprised 901 middle schoolers recruited from three middle schools, two of which were public schools and the third a private school, in an urban area in China. In China, there are no nationally used indicators of either individual or school level socio-economic status such as percentage of students eligible for free school meals which are commonly used in Western studies. Thus, a private school, where tuition fees are levied, was included to increase sample diversity. The data was collected during the first semester of the 2019 academic year. Eighty-three students were excluded from the main analysis because they either chose not to be included or they admitted giving careless responses. Therefore, the final data contained 818 (45.9% girls) eighth graders, who ranged in age from 12 to 15 years (M = 13.39, SD = 0.54). Age differences vary due to different admission ages, repeating or skipping a grade, or other reasons of delay.

This study received institutional ethical approval and followed the British Educational Research Association (BERA) guidelines. Permission was obtained from the school administration in the first instance, who then informed parents about the study. Assent was then obtained from the students themselves. Written and verbal instructions informed participants that their answers would remain confidential and that they could withdraw from the study at any time. The students were asked to complete the survey during their mental health classes, which lasted about 45 mins. Records of grades were requested directly from teachers after the data collection. The data collection period was from October to November, which coincided with the timing of mid-term exams.

### Measures

#### Peer nomination

Peer nomination procedures followed standard approaches to assessing prosocialness, aggression, and social status among Chinese middle schoolers (Cillessen andMayeux 2004); each area was assessed using two questions, totaling six altogether. The order of these six peer-nominated questions was randomized. Each participant was given a roster of all their classmates’ names with code numbers and were asked to write down all the names of the students they felt fitted the given description. The study contends that peers could give accurate judgments based on their classmates’ overall manners because they remain in the same administrative classes for a period of three years (Niu et al., 2016). Empirical studies suggest that when unlimited nomination procedures are used, and the response rate for nominations is greater than 60%, the internal consistency will significantly increase (Marks et al., 2013). In this study, self-nominations were removed when calculating the scores, and unlimited nominations for each item were allowed. The participation rates in different classes ranged from 73.5–97.9%. The participant’s score for each question was calculated as the number of nominations a participant received over the total number of participants in each class, and the scores were then z-standardized for further comparison. This ensured that differences in class size would not affect the proportion of nominations a student received.

#### Prosocial behavior

Participants were asked to nominate two prosocial descriptors separately: “someone who follows the rules and cooperates with others” and “someone willing to help others when they need it”. These two items are derived from the Revised Class Play scale (Masten et al., 1985), and have been successfully translated for use with Chinese adolescents (Chen et al., 2000); both studies demonstrated good reliability of the two subscales (*α* = 0.89−0.92).

#### Aggressive behavior

Aggressive behavior was measured using two aspects—physical aggression (kicks, pushes, or hurts others) and relational aggression (tells lies or spreads false rumors about others) – which were also modified from the Revised Class Play (Masten et al., 1985). Aggressive indicators have also been widely used in a Chinese context and demonstrated good reliability respectively (*α* = 0.94 –0.96) (Tseng et al. 2013).

#### Social status

Participants’ popularity and likeability were utilized to assess participants’ social status, with participants being asked to nominate students in line with two standard statements: “the students who are the most popular in your class” and “the students you like the most in your class” (Cillessen and Mayeux, 2004). A previous study found the two items were highly correlated in Chinese youth (*r* = 0.75) (Zhang et al., 2018) and a similar result was found in the current study (*r* = 0.63).

**Fig. 1:**
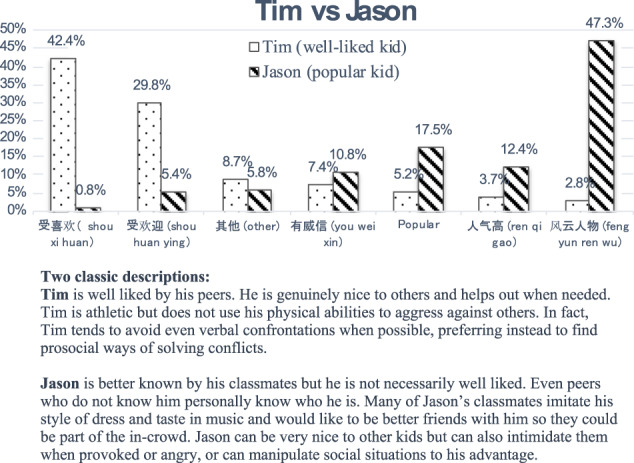
Outcomes of preferred translations for the descriptions of Tim and Jason

Given inconsistent translation of the term “perceived popularity” in the Chinese literature, a pilot study was carried out before the main study to determine the most appropriate translation. First of all, with the assistance of several bilingual speakers and based on existing literature (see below), six commonly used translations of popular were identified (see Fig. 1). Next, two classic descriptions of being liked (Tim) and being popular (Jason), as shown in Fig. 1, were adopted from an existing study (Cillessen and Rose, 2005). These descriptions were subsequently presented to participants alongside the six commonly used translations of popular. Participants were asked to choose which of the translations firstly best described Tim and secondly best described Jason. Participants were a convenience sample of Chinese students studying in the UK recruited via social media (N = 248). Ten participants were excluded due to inattentive answers. “Ren qi gao” and “feng yun ren wu” are two recommended translations to supplement the conventional translation of popularity “shou huan ying” (Niu et al., 2016). From the results, it is clear that “shou huan ying” should not be used as a gold standard translation of popularity since the meaning of it overlapped with likability to a great extent. This can be seen from the fact that “shou huan ying” was also the second ranked choice (29.8%) to describe the well-liked child Tim. It appears that “feng yun ren wu” is superior to “ren qi gao” as a translation of popular, not only because it was identified by almost half the sample (47.3%) but also because it separates itself to a great extent from likeability as only 2.8% of participants indicated this best described Tim. This finding, while preliminary, suggests “shou xi huan” and “feng yun ren wu” may best capture the nuanced meanings of being liked and being popular and discriminating between them.

#### Self-perceived socioeconomic status

The current study adopted a youth-suitable version of the MacArthur Scale of Subjective Social Status (MacArthur SSS Scale), which has good reliability and validity in measuring an individual’s perception of their socio-economic status (Adler and Stewart, 2007; Goodman, 2001). The scale presented a ten-rung ladder on which participants were asked to rank their family’s socioeconomic level, corresponding to the numbered box within the ladder. This measurement has a significant test–retest reliability of 0.62 (Operario et al., 2004) and has been used previously when researching the Chinese population (Hu et al., 2005).

#### Academic achievement

Chinese, Mathematics, English, and Science are the four core subjects in Chinese schools across all provinces, thus these were used collectively to assess academic achievement, as this a better indicator than a one-subject statistic (Sirin, 2005). Scores were derived from the latest mid-term exams with higher scores indicating better grades. Raw scores in each core subject were standardized within each class for comparability as each school has its own scoring system, and the students’ final academic achievement score was their summated standardized scores across the four subjects.

#### Psychological wellbeing

The psychological wellbeing of participants was assessed using the Flourishing Scale (Diener et al., 2009). This measure comprises eight items (e.g., “I am optimistic about my future”, “I lead a purposeful and meaningful life”), each of which is rated on a seven-point Likert scale varying from 1 (“strongly disagree”) to 7 (“strongly agree”). A high score indicates that a person has higher psychological wellbeing and strengths. This scale has been validated in Chinese adolescents and demonstrated a high internal consistency (*α* = 0.83) (Duan and Xie 2019).

### Analytical Strategy

#### Missing data

A range of 0.2–0.7% of reports was missing. Little’s MCAR test revealed that the missing data was completely missing at random (*x*^2^(105) = 118.45, *p* = 0.17). Missing values in SPSS were replaced with multiple imputations using an EM algorithm. Missing values in Mplus were addressed by the Full Information Maximum Likelihood (FIML), using all available data to maximize the information.

#### Latent profile analysis

Analysis used Mplus 8 (Muthén and Muthén, 1998–2018), and applied the maximum likelihood estimator with robust standard errors (MLR) (Muthén, 2004) to conduct a cross-sectional latent profile analysis to identify participant subgroups. This person-centered approach determined latent profiles that have more homogenous response patterns (Muthén and Muthén, 2000). Latent profile analysis was utilized to study social status types of the adolescents based on six variables (physical aggression, relational aggression, help, cooperation, popularity, and likability); all six were assessed using standardized units to facilitate interpretation.

The best-fitting model was selected based on guidance from several criteria. The following statistical indicators were assessed: entropy; Akaike information criterion (AIC); the Bayesian information criteria value (BIC) (Nylund et al., 2007); the adjusted BIC (aBIC); the Lo, Mendell, and Rubin likelihood ratio test (LMR-LRT) (Lo et al., 2001); and the bootstrapped likelihood ratio test (BLRT). A high level of entropy indicates a greater accuracy of classification (Jung and Wickrama, 2008). A lower level of AIC, BIC, and aBIC suggests a better fitting model (Nylund et al., 2007). Moreover, LMRT and BLRT were used to compare the k-1 versus k class model, with significant values of LMRT and BLRT

supporting the latter. Once the number of profiles had been determined, ANOVAs were conducted in SPSS (IBM SPSS Statistics 23) to explore how profiles differed from one another. Prior to an analysis of variance, Levene’s test revealed that the variances of physical aggression, relational aggression, likeability, and the popularity dataset were not equal (F (3814) = 30.32–111.88, *p* < 0.001), so alternative Welch’s statistics were adapted to determine the significance.

Finally, the predictors and outcomes of latent profiles were assessed using the R3STEP method and the BCH method in Mplus. Specifically, the R3STEP command was used to conduct multinomial logistic regressions to examine the effect of gender, school attended and self-perceived SES on predicting a student’s likelihood of belonging to specific groups. Moreover, the BCH method was performed as a weighted ANOVA in Mplus to examine the differences in students’ mental health and learning outcomes across profiles.

## Results

### Preliminary Analyses

Descriptive statistics and bivariate correlations for the key variables are presented in Table 1. Popularity and likeability were positively correlated, as were the two aggressive behaviors (physical aggression and relational aggression) and the two prosocial behaviors (cooperation and help). Prosocial behaviors (cooperation and help) were positively correlated with social status, particularly likeability. Aggressive behaviors (relational and physical) were negatively correlated with likeability and had no association with popularity. Moreover, aggressive behaviors and prosocial behaviors were negatively correlated.

**Table 1:** Descriptive statistics and bivariate correlations for key study variables

	Physical aggressive	Relational aggressive	Cooperation	Help	Popularity	Likeability
Physical aggression	–					
Relational aggression	0.63**	–				
Cooperation	−0.34**	−0.36**	–			
Help	−0.29**	−0.24**	0.81**	–		
Popularity	0.04	0.06	0.42**	0.46**	–	
Likeability	−0.26**	−0.21**	0.75**	0.76**	0.63**	–
M	1.98	3.27	17.35	16.92	5.03	10.38
SD	4.13	4.54	8.63	7.54	6.38	7.44

*p < 0.05, **p < 0.001

### Social Status Profile Identification

A four-group model was selected as the optimal solution. Table 2 presents the latent profile analysis fit indices for up to five profile groups. The four- and five-profile group models displayed lower AIC, BIC, and ABIC when compared with the previous one-to-three solutions. BLRT indicated that the five-profile model was superior to the four-profile model, whilst the LMR suggested the opposite (*p* = 0.183). Comparing groups of the four- and the five-profile models, the first four groups remained stable, and the fifth group only contained 1.8% of total participants. Theoretically, the four-group model was more apt than the other models. Thus, the four-group model was selected for better interpretability. The four average latent class probabilities were 0.92, 0.98, 0.92, and 0.97, which indicated that the groups are clearly differentiated from each other. Although the entropy value (0.87) of the four-group solution was the lowest among the five solutions, it was still good enough to conclude that the four-group solution delivered a precise classification (Muthén and Muthén 2000).

**Table 2:** Model fit indices for the latent profile classification with 1-5 classes

Profile	AIC	BIC	aBIC	*p*LMR	*p*BLRT	Entropy	Group
1	13946.30	14002.78	13964.67	–	–	1	818
2	12631.30	12720.73	12660.39	0.000	0.0000	0.90	645/ 173
3	11654.14	11776.52	11693.95	0.016	0.0000	0.94	606/161/51
**4**	**11163.62**	**11318.94**	**11214.15**	**0.002**	**0.0000**	**0.87**	**342/325/103/48**
5	10781.34	10969.62	10842.59	0.183	0.0000	0.89	328/307/103/65/15

Values in bold type indicate the chosen model in this study

*AIC* Akaike information criterion, *BIC* Bayesian information criterion, *aBIC* the adjusted Bayesian information criterion, *LMR* Lo, Mendell, and Rubin likelihood ratio, *BLRT* bootstrapped likelihood ratio test

**Fig. 2:**
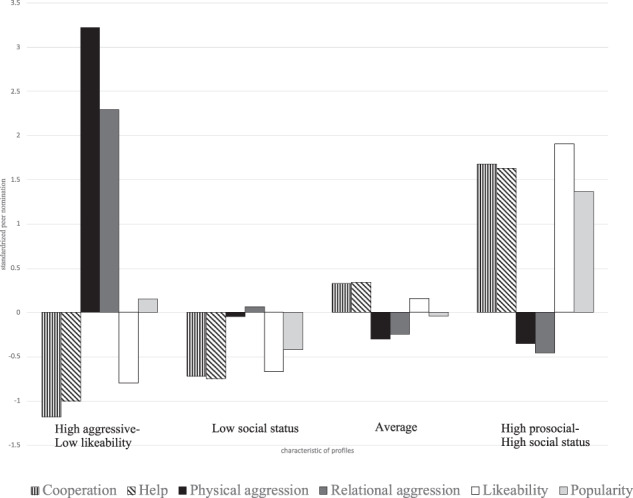
Characteristics of profiles by their standardized peer nomination score (N = 818)

All variables were converted to z-standardized scores for easier comparison and interpretation. To examine the differences between four profile groups, which will be elucidated below, on internalizing indicators (cooperation, help, physical aggression, relational aggression, popularity, and likeability), a series of ANOVAs were conducted. The mean differences of key variables between different profiles can be found in Table 3. As shown in Table 3, the post hoc analyses (LSD) revealed that early adolescents in different profiles had significant differences in their classification variables, and these are illustrated in Fig. 2.

**Table 3:** Mean differences of key variables between different profiles M(SD)

	High aggressive-low likeability	Low social status	Average	High prosocial-high social status	F
Cooperation	−1.1 (0.45)_d_	−0.74 (0.55)_c_	0.35 (0.55)_b_	1.69 (0.50)_a_	737.38**
Help	−0.99 (0.53)_d_	−0.76 (0.57)_c_	0.37 (0.55)_b_	1.64 (0.60)_a_	556.25**
Physical aggressive	3.24 (1.56)_a_	−0.03 (0.56)_b_	−0.32 (0.34)_c_	−0.34 (0.29)_c_	103.07**
Relational aggressive	2.31 (1.50)_a_	0.08 (0.82)_b_	−0.27 (0.70)_c_	−0.46 (0.48)_d_	67.79**
likeability	−0.79 (0.43)_c_	−0.69 (0.39)_c_	0.18 (0.61)_b_	1.93 (0.73)_a_	515.52**
Popularity	0.16 (0.96)_b_	−0.43 (0.56)_c_	−0.04 (0.80)_b_	1.40 (1.37)_a_	70.84**

Values with different subscripts in the same row are significantly different from one another based on the post hoc analyses (LSD)

*p < 0.05, **p < 0.001

Profile 1—high aggressive-low likeability group (N = 48)—represented 5.9% of the total sample. This aggressive group exhibited significantly high levels of aggressive behaviors and extremely low levels of likeability and prosocial behaviors and scored second highest (along with the average group) on popularity. Profile 2 represented 39.7% of the total participants and was labeled as the low social status group (N = 325), as they embodied the lowest (along with the high aggressive group) likeability and the lowest popularity. Students in this group performed the second-highest levels of physical aggression and relational aggression and the second-lowest levels of cooperation and helpful behavior.

Profile 3 was characterized as the average group (N = 342), representing 41.8% of the total sample. This group’s participants had the lowest difference in means across the six variables, making them “average” among their peers. The average group scored the second-highest overall on prosocial behaviors and significantly below the sample mean on aggression. Their level of popularity was not significantly different from the aggressive popular group, but their likeability was the second highest among the four groups. Profile 4—the high prosocial-high social status group (N = 103)—represented 12.6% of the sample. This group scored highest on both likeability and popularity and had the highest level of prosocialness and the lowest level of physical aggression (along with the average group) and relational aggression.

Table 4 presents the effects of gender, school attended (public vs private), and subjective social status on predicting a student’s profile membership using multinomial logistic regressions. Chi-square analyses indicated that gender differences, school attended differences, and subjective social status differences in profile membership were significant, (*x*^2^(9) = 45.88, *p* < 0.0001; *x*^2^ = (45), *p* < 98.83, *p* < 0.001; *x*^2^(3) = 35.29, *p* < 0.0001).

**Table 4:** Coefficients for the four profiles with gender, school attended and SSS as predictors

	Low social status vs Aggressive		Low social status vs Average		Low social status vs High social status		Aggressive vs Average		Aggressive vs High Social status		Average vs High social status	
	logit	odds	logit	odds	logit	odds	logit	odds	logit	odds	logit	odds
Gender	−2.45***	0.09	0.59**	1.81	0.42	1.53	3.04***	20.98	2.87***	17.67	−0.17	0.84
School	0.56	1.76	0.62*	1.86	1.45***	4.25	0.06	1.06	0.89*	2.42	0.83*	2.29
SSS	0.03	1.02	0.22**	1.24	0.39***	1.47	0.20	1.21	0.36*	1.43	0.17	1.18

*p < 0.05, **p < 0.001, ***p < 0.0001

The comparison of the aggressive group, the average group, and the high social status group with the low social status group (as a reference group) revealed that the private school students were more likely to fall into the average or high social status group than students who attended public schools (B = 0.62, *p* < 0.05, odds ratio [OR] = 1.86; B = 0.1.45, *p* < 0.0001, OR = 4.25); similarly, students with a high level of subjective social status predicted an increased likelihood of being placed in either the average or high social status group (B = 0.22, *p* < 0.001, OR = 1.24; B = 0.39, *p* < 0.0001, OR = 1.47). When comparing the high social status group relative to membership of the aggressive group, the results indicated that the private school students had a higher possibility than the public schools’ students to fall into the high social status group (B = 0.89, *p* < 0.0001, OR = 2.42), and students with higher self-perceived SES were also associated with increased odds of belonging to the prosocial group rather than the aggressive group (B = 0.36, *p* < 0.05, OR = 1.43). Finally, when using the average group as the reference group, the private school students were more likely than the students from public schools to fall into the prosocial group (B = 0.83, *p* < 0.05, OR = 2.29).

In terms of gender, the results revealed that 94% of the aggressive group were boys but that the gender distribution of the other three groups was more balanced. Table 4 indicated that girls were more likely than boys to fall into the low social status group rather than the aggressive group (B = −2.45, *p* < 0.0001, OR = 0.09). Compared to girls, boys were more than ten times as likely to be in aggressive group, rather than prosocial and average groups (B = 2.87, *p* < 0.0001, OR = 17.67; B = 3.04, *p* < 0.0001, OR = 20.98). Moreover, when using the average group as the reference group, the results indicated that boys were more likely to fall into the low social status group than girls (B = 0.59, *p* < 0.001, OR = 1.81).

Table 5 displays the mean and SD of the wellbeing and learning outcomes across all four profiles. Profiles had a main effect on psychological wellbeing and academic achievement (F (3814) = 13.832, *p* < 0.0001; F (3814) = 48.75, *p* < 0.0001). As shown, the psychological wellbeing of the average and prosocial groups was significantly higher than those in the low social status and aggressive groups. Notably, there were no differences between the wellbeing of low social status adolescents and aggressive adolescents. Moreover, those in the prosocial group exhibited the highest level of academic achievement, whereas the low social status and aggressive students obtained the lowest academic achievement.

**Table 5:** Differences between four profiles on wellbeing and learning outcomes M(SD)

	High aggressive-low likeability	Low social status	Average	High prosocial-high social status
Psychological wellbeing	40.27 (1.03)_b_	40.86 (0.51)_b_	45.45 (0.40)_a_	46.70 (0.73)_a_
Academic achievement	−0.51 (0.17)_c_	−0.41 (0.06)_c_	0.23 (0.05)_b_	0.74 (0.07)_a_

Values with different subscripts in the same row are significantly different from one another based on the BCH method

## Discussion

Prosocial and aggressive behavior have long been researched in social status studies, however, the nature of the relationships between these behaviors and social status has remained unclear. Due to the complexity and interdependency of these variables, a latent profile analysis study may to some extent offer a new perspective on these relationships. The current study aimed to identify the naturally existing social status groups in China in early adolescents and to determine whether prosocial and aggressive behavior could co-exist within a social status profile. Furthermore, it aimed to explore the associations between profiles and gender, school attended, self-perceived SES, academic achievement, and psychological wellbeing to further characterize these profiles. Using latent profile analysis, four unique social status profiles were identified, and their prevalence was documented. Moreover, these profiles not only had significantly different social status characteristics, but also different patterns of associations with the above-mentioned variables making it possible to characterize them more fully. As predicted, the study failed to locate a bi-strategic group in the Chinese sample, but it did discover a low social status group that had not been uncovered in the Western literature which is discussed further below.

The study identified approximately 6% of the sample as comprising the high aggressive group. It should be noted that the name of each group is not intended to pejoratively label these students but rather aims to draw attention to their distinct qualities. Peers seldomly identified students in this group as performing prosocial behaviors but always signaled they behaved aggressively. These aggressive students were not well-liked by their peers but were somewhat (higher than the low social status group and same as the average group) popular in their classes. Although aggressive students with a low level of likeability have been found in both variable and person-centered studies, previous studies have been inconsistent about the prevalence of this aggressive popular group: one found under 5% of the sample fitted this description, whereas the other found around 15% fitted it (Hartl et al., 2020; Berger et al., 2015). This may come from the different indicator choices as illustrated in the introduction. Other than this, differences in prevalence may be explained by the different levels of cultural endorsement of aggressive behaviors in different countries. Countries which value physical power or emulation would be more likely to have a higher level of tolerance for their adolescents performing this type of behavior (Cillessen and Mayeux, 2004), which may lead to a higher prevalence of aggressive teenagers.

The low social status profile as characterized in this study does not appear to have been found in previous research. This group, representing around 40% of the sample, was the only group identified with below-average scores on both popularity and likeability, indicating that they were left out by their peers when nominating popular and likeable students. The average group also contained approximately 40% of the sample and was also characterized by the second-highest level of prosocial behavior, the second-lowest level of aggression, and an average level of social status. Thus, the low social status and average groups significantly differed in their aggression, prosociality, and social status levels and had significantly different associations with all predictors and outcomes, further discussed below. Therefore, there is no doubt that these two groups are distinct, at least in this sample. However, previous latent profile analysis studies have failed to discover this low social status group and instead have classified around 65–75% of their samples in the average group. It is therefore plausible that previous studies might have aggregated these two groups within the average group, but more research is needed to validate this speculation.

This newly found low social status group resonates with the “neglected group”, which has been categorized in sociometric status studies as a group of children with low visibility who are neither liked nor disliked by their peers, when deploying a statistical criterion of the nominated social preference and social impact (van der Wilt et al., 2018). The current study could not rule out the possibility that low social status students were also not disliked by their peers as no data on disliked children were collected due to ethical concerns. It is conceivable that the low social status group may contain some neglected students. However, several other characteristics would suggest that the low social status group is different from the neglected group. First, the neglected group has been found to be less aggressive than the average group (van der Wilt et al., 2018) whereas low social status students in this study had a significantly higher degree of aggression than those in the average group. Second, some researchers have indicated that the advent of the neglected group may be due to the use of a limited nomination procedure which might boost the number of children in this group (Terry, 2000). On average, the neglected group was found to encompass 9% of the population. However, the present study applied an unlimited nomination technique and found a particular predominance of students (40%) in this specific group. Third, previous sociometric status studies suggested that the neglected group was not at risk of developing negative outcomes due to its unstableness and similarity to the average group. However, this study uncovered the low social status group performed as a moderate version of the high aggressive group and is at high risk of having low wellbeing and low academic achievement; details will be illustrated in the below section. Thus, it is possible that the low social status group and the neglected group may share some nuanced similarities but overall the evidence suggests they are two distinct groups.

If the low social status group is unique in Chinese schools, the typical class size in China might be an alternative explanation to account for its presence. The class sizes and student-faculty ratio in Chinese junior high schools are much larger than in Western middle schools.

The average class size in the two participating public schools was 47. Such a large class size makes it difficult for teachers to track each student’s development and give them enough attention (Beattie and Thiele, 2016). If those students who develop slowly in their studies and social skills are not supported by their teachers promptly, they may be considered unvalued or unattractive to study or associate with by their peers and may gradually develop maladjusted strategies to cope with this situation. Ultimately, these quiet and perhaps a little reserved students may not have been given adequate attention, and thus formed this low social status group. More investigation to scrutinize this group is needed.

Last, the high prosocial-high social status group encompassed around 13% of the sample and was underlined by its remarkably high prosocial performance, low aggression, and unanimous acceptance and social dominance. All prosocial groups in previous studies, alongside the current study, confirmed this group of adolescents had the highest likeability among their peers. However, in contrast to Western studies, the high prosocial group in China in this study also had the highest popularity. When the prosocial group was compared with the average group, this indicated the absence of aggressive behaviors alone is insufficient to achieve full-scale (both likeability and popularity) high social status; rather it must be accompanied by a high level of prosocial behaviors. As Chinese students grow older, so does their intense academic burden. Moral courses that are not included in the college entrance examination often give way to more “important” courses, such as Mathematics, Science, or Chinese. These results should encourage teachers to incorporate prosocial education into their classrooms and could serve as a reminder of the need to increase the weighting of prosocial education in the curriculum in Chinese schools.

Finally, the bi-strategic group was not found in the present study as no profile contained both prosocial and aggressive behavior above the sample mean. A supplementary analysis only deploying the more limited profile indicators of a key previous study (e.g., only popularity, aggressive behavior, and prosocial behavior, Hartl et al., 2020) to aid comparability, was able to confirm the average popular, prosocial popular, and aggressive popular group, but still failed to reveal a bi-strategic popular group. Thus, there was not a group of students in this sample who performed both prosocial and aggressive behavior. Like the Master said, ‘If the will be set on virtue, there will be no practice of wickedness.’ (Confucius and Legge, 2008). Confucianism, as the foundation for Chinese culture, places great emphasis on the importance of maintaining group wellbeing and harmony, whereas the self-interested nature of aggressive behaviors could potentially harm group stability and lead to individuals becoming loathed and rejected (Zhang et al., 2020), and this may lead Chinese students to be less likely to perform both prosocial and aggressive behavior. Thus, the failure to discover a bi-strategic group may not result from choosing different profiles’ indicators but primarily be attributed to cultural differences. This is the first study to test the bi-strategic group in a collectivist context using a person-centered approach, thus further investigation is still needed.

As theoretically hypothesized in resource control theory, this study was consistent with previous studies in which boys outnumbered girls in the aggressive group (Hartl et al., 2020). Moreover, compared with girls, boys were more likely to be in the aggressive group than in the prosocial, low social status, or average groups. When using the average group as the reference group, the results indicated that boys were more likely to fall into the low social status group than girls. As the low social status and aggressive groups were associated with the lowest psychological wellbeing and academic achievement, this finding suggests boys are more vulnerable to falling into the two more maladaptive groups. The findings echo the literature on boy’s low achievement (Yu et al., 2020). Thus, future research could explore the cause with the aim of understanding how this disadvantageous development can be prevented.

Private schools in China generally require higher tuition than public schools to maintain better school facilities which explained why school type and subjective social status shared the same pattern of predicting the distribution of profile membership. The results revealed a higher subjective social status or attending a private school predicted an increased likelihood of being in the average and prosocial groups, the two more adaptive profiles among the four profiles. Given subjective SES is a medium-to-strong factor which could potentially affect one’s psychological wellbeing (Quon and McGrath, 2015), prosocial behavior (Cowell et al., 2017), and academic achievement (Sirin 2005), it is not surprising that students in the prosocial group had the highest academic achievement and psychological wellbeing. This aligns with numerous previous findings, which showed that prosocial behaviors predict later academic achievement and happiness (Park et al., 2017; Diener and Seligman, 2002). Moreover, previous latent profile analysis studies found the prosocial popular profile predicted the lowest level of negative externalizing behaviors (Hartl et al., 2020), and a social emotional-prosocial profile is associated with the highest academic achievement (Collie et al., 2019). Again, prosocial behavior seems a strong indicator of academic achievement and wellbeing, which is testified in both variable-centered and person-centered studies.

However, it is also important to highlight that there is no need to encourage all students to become prosocial group students, particularly if they are average students who are satisfied with their standing. Compared with the prosocial group, the average group had a significantly lower score on academic performance but showed no difference in psychological wellbeing. A study found the relationship between both dimensions of social status and social contentment is nonlinear (Ferguson and Ryan, 2019), which may explain this outcome. Understandably, to maintain high social status, these students need to make a considerable investment of effort and monitoring, which could undermine their psychological wellbeing (Allen et al., 2005). This was also confirmed in previous findings where the prosocial popular group was not superior in terms of their level of anger and disruptiveness than the average group (Hartl et al., 2020). Thus, the finding suggested that although prosocialness is beneficial to both performers and receivers (Weinstein and Ryan, 2010), there is no need to spur all students to become perfectly behaved students in terms of prosocial acts. Approaches which either emphasize tough instruction or very minimal intervention are both dogmatic. Instead, teachers should guide students based on their individual needs. Thus, cultivating adolescents to achieve a balanced amount of prosocialness could be a possible adjustment that teachers can implement in their classes.

It should be noted that the high aggressive and the low social status groups had the lowest academic achievement and psychological wellbeing. Until now, research has been unclear about whether aggression has the same adaptive functioning as prosocial behaviors, or whether its positive effects come from the association with being recognized among peers (Berger et al., 2015). Although the high aggressive students in this study were popular to a certain extent, as this was also associated with the lowest academic achievement and wellbeing, it is hard to believe that aggression is adaptive in a Chinese context. The high aggressive group has long received extensive attention; thus, the study highlights the particular need to pay greater attention to low social status adolescents as they also share the same vulnerability as the aggressive group and even had a lower level of popularity. It is worth bearing in mind that the low social status group in this study is a mild version of the aggressive popular group since the youth in this group also had low prosociality (the second lowest) and high aggressiveness (the second highest). According to social learning theory (Hoorn et al., 2016), it is possible that some of these students who already have a propensity towards aggression, would increase their aggressiveness, learning from their aggressive popular peers, to become more popular. Thus, it matters that teachers recognize such students and acknowledge their needs to facilitate them in preventing such maladaptive behavioral development.

Although this study contributes knowledge of social status profiles and their associations with gender, socioeconomic status, school attended, academic achievement, and wellbeing, several limitations could be addressed in future research. First, several other aspects of the profiles’ indicators were not included in this study. For example, the functions of aggression (proactive and reactive) and verbal aggression were not considered. Researchers have found that popularity has opposite correlations with proactive and reactive aggression (Stoltz et al., 2016). Moreover, likeability and popularity are the two most dominant forms of youth’s high social status (Cillessen and Rose, 2005), but a new suggested form, admiration, has been less well studied (Zhang et al., 2018). Therefore, future research could explore how including the aforementioned indicators might result in slightly differing social status profiles.

Furthermore, it is important to recognize certain methodological limitations. First, the data were collected only through peer nomination techniques. A future study could consider integrating both self- and teacher-reported data to testify the outcomes. Second, this study is culturally specific as it only pertains to Chinese middle schoolers, but the findings are positioned in relation to resource control theory making it comparable with previous studies. Thirdly, the cross-sectional nature of the data could account for some of the differences found compared with previous longitudinal work. A longitudinal study may also be able to shed light on the causal relationships between social status profiles and subsequent academic achievement and wellbeing, which cannot be determined in this study.

## Conclusion

Previous research that has studied the relationships between prosocial and aggressive behavior with likeability and popularity deploying a variable-oriented approach, and earlier person-oriented studies, have not been consistent concerning the nature of natural social status profiles, especially in relation to a bi-strategic group hypothesized by resource control theory. Thus, the current study adopted behavioral and social status indicators using a large Chinese sample. While it failed to confirm the bi-strategic group, it nonetheless discovered a low social status group along with a high aggressive-low likeability group, an average group, and a high prosocial-high social status group. By using a person-centered approach, this study was able, to some extent, to untangle the relationships between prosocial, aggressive behavior with social status, and found that to obtain a high social status in China, both a high level of prosocial-ness and a low level of aggression are required. Furthermore, patterns of associations with gender, self-perceived SES, school attended, academic achievement, and psychological wellbeing were studied to better understand each group. Whilst patterns of associations further characterized the profiles and suggested these were generally comparable to those found in studies conducted in Western contexts, there were several notable departures that extend previous findings. The low social status group was uniquely characterized as exhibiting the lowest academic achievement and wellbeing comparable to their high aggressive peers and given the similarities between these profiles this suggests that more attention should be devoted to these students both in school and in future research. Overall, the study findings build upon and extend the predominantly Western research on adolescents’ peer relations in a non-Western domain.
